# Kinematic Analysis of Free Vertical Split with 720° Turn in Elite Chinese Rhythmic Gymnastics

**DOI:** 10.3390/s25092667

**Published:** 2025-04-23

**Authors:** Tao Liu, Liangsen Wang, Liquan Gao, Yuliang Sun

**Affiliations:** School of Physical Education, Shaanxi Normal University, Xi’an 710119, China; liutao0604@snnu.edu.cn (T.L.); wlsen13@snnu.edu.cn (L.W.)

**Keywords:** kinematic, rhythmic gymnastics, sports injury, joint angle, center of mass

## Abstract

This study investigates the kinematic characteristics of the free vertical split with 720° turn (C 807). C 807 is the international designation in rhythmic gymnastics for a free vertical split with a 720° turn. This research holds significant importance in enhancing the technical proficiency of gymnasts and reducing their risk of injury. Eight national-level female gymnasts (age = 20 ± 3 years) performed the C 807. Kinematic data were collected using a 3D motion capture system. The movement was divided into four phases, and Visual 3D (V6.0, CMotion, Germantown, MD, USA) software was used for data processing and analysis. The joint angles of the upper and lower limbs, as well as the torsion angles of the lower limb joints, were analyzed. Key findings included tibial torsion, knee hyperextension in the support leg, and changes in elbow flexion during each phase. The center of mass (COM) trajectory showed that, during the backward preparatory swing phase, COM height gradually decreased and slightly increased before the initiation phase. In the initiation phase, COM height initially decreased and then increased, while the rotation phase showed fluctuating but stable COM height. The results highlight the importance of joint angle control and COM fluctuations during movement. Training should focus on leg swing speed, lower limb strength, knee stability, and upper limb coordination to enhance balance, improve rotation speed, and prevent injuries.

## 1. Introduction

The free vertical split with 720° turn (C 807) is a classic rotational movement in rhythmic gymnastics (RG), frequently appearing in competitions and serving as a key element for scoring. RG is an aesthetic discipline at the intersection of art and sport [1]. Combining athleticism and beauty, RG seamlessly integrates artistic expression with competitive movements, creating a visually captivating and technically demanding sport. Over the past thirty years, the complexity and diversity of performance techniques in RG has increased significantly, driving rapid advancements in the sport. This evolution has been fueled by rising levels of competition, higher participation rates, and the growing creativity of gymnasts. At the same time, advancements in sports science particularly in sports biomechanics have played a pivotal role in enhancing performance and refining techniques [2]. Despite these advances, the longstanding use of the same apparatus for approximately 70 years, coupled with the increasing intensity of the sport, have contributed to a higher risk of injuries. Studies show that lower limb injuries are particularly prevalent among rhythmic gymnasts, with ankle injuries accounting for 33.3%, followed by knee injuries (18.8%) and Achilles tendon injuries (14.6%) [3]. Another study highlighted a relatively high injury rate in the hip joint among gymnasts [4]. Movements, such as C 807, impose substantial loads on the lower limbs and lower back, resulting in a high incidence of pain and injury. These challenges are particularly pronounced in gymnasts performing at advanced levels [5]. To address these concerns, scientific training methods and proper techniques are essential. By optimizing movement patterns and biomechanical efficiency, athletes can enhance their performance and reduce the risk of injury, contributing to the sport’s continued growth and the well-being of its participants.

Despite gymnastics’ widespread popularity, there is limited research exploring the biomechanics of its diverse skills or the relationship between movement techniques and athletic performance. The high biomechanical demands of gymnastics emphasizes the need for a deeper understanding of movement techniques to guide training and reduce injury risks [6]. While some biomechanical studies in gymnastics have focused on jumping movements, such as the takeoff and landing characteristics of somersaults, these investigations have often revealed angular momentum differences across skill variations [7]. Other research has examined specific elements, such as the influence of three distinct arm swing techniques on the elevation of the center of mass during a standing backflip, as well as the biomechanical responses to various landing strategies [8,9]. Beyond jumping, studies are expanding into other key skills. For instance, straight-arm handstand techniques have been analyzed to determine kinematic profiles and joint moment distributions [8]. Similarly, investigations into front handspring support techniques have provided insight into the impact loads during vaulting [9]. Although these studies have provided valuable insights into specific movements, a notable gap in research remains regarding the biomechanics of rotational movements in gymnastics. Expanding research into this area could further enhance the understanding of gymnastics techniques, benefiting athlete performance and safety.

This study examines the kinematic characteristics of the C 807 movement in female rhythmic gymnasts, quantitatively evaluating movement patterns to identify techniques that optimized performance and reduced injury risks. Based on the hypothesis that this skill increases injury susceptibility, the analysis focuses on lower limb joint angles to provide insights into biomechanical demands and inform preventive strategies.

## 2. Materials and Methods

Given that elite athletes’ movement patterns converge toward biomechanical optima and national-team-level rhythmic gymnasts are exceptionally rare, this study adopted a small-sample case study design—a methodology previously employed in technical movement analysis of elite rhythmic gymnasts [10,11]—focusing on movement characterization rather than group comparisons.

### 2.1. Participants

Eight female rhythmic gymnasts (age: 20 ± 3 years; height: 1.61 ± 0.01 m; weight: 47.1 ± 2.7 kg) participated in this study. All participants were elite athletes from the national team and the reserve team. The inclusion criteria required that the athletes had suffered no lower limb injuries in the past three months, including any injury that could impair athletic performance, as well as no neurological conditions that might affect their gymnastics performance. After providing a detailed explanation of the study’s purpose and procedures, informed consent was obtained from all participants. The Shaanxi Normal University ethics committee approved the study (202416044).

### 2.2. Specific Action Description

**Figure 1:**
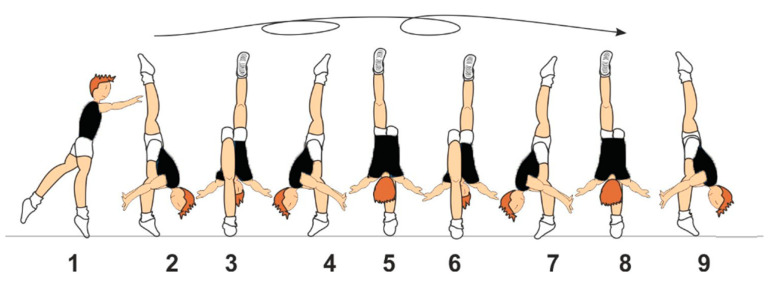
The gymnast starts by standing on one leg (1), transitions into the free vertical split with the non-supporting leg raised (2–3), and then performs a 720° (2/1) rotation (4–9), maintaining the supporting leg vertically on the floor throughout the movement.

The technique is a free vertical split with 2/1 turns (C 807), combining a standing position on one leg, the split, and a two-and-a-half turn (720°) rotation. According to the minimum requirements, all elements in Family VIII (Family 8: Flexibility) must not be performed through individual movements, the angle between the legs must be at least 170°, and the supporting leg must be in a vertical position, not leaving the floor (Figure 1).

### 2.3. Apparatus and Procedures

**Figure 2:**
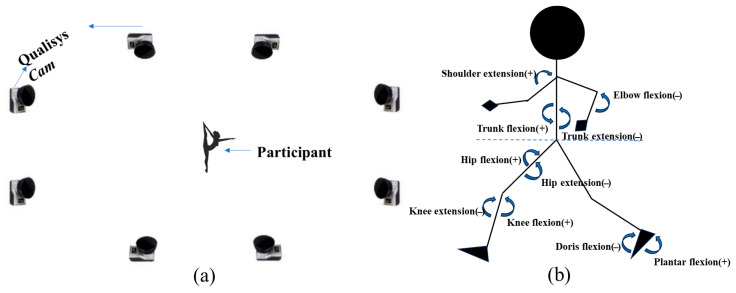
(**a**) Experimental setup of the study; (**b**) the sagittal plane model of the trunk, lower, and upper limbs.

The experiments were conducted in the university’s biomechanics laboratory, which was maintained at an appropriate temperature. The laboratory dimensions were approximately 10 m in length, 5 m in width, and 3 m in height (Figure 2a). Throughout all tests, the gymnasts wore the same style of tights. After a standard 15 min warm-up, participants practiced the movements in a regional center to ensure they were familiar with the process. Three valid trials were conducted for each test, and the average values of these trials were used to ensure the accuracy of the experimental data.

### 2.4. Kinematics

A motion capture system consisting of eight infrared high-speed cameras (Oqus 700+, Qualisys AB, Gothenburg, Sweden, 200 Hz) was used to collect kinematic data. We selected the Qualisys motion capture system based on its established reliability in peer-reviewed biomechanics research [12,13]. The Qualisys infrared high-speed camera commonly utilizes a CMOS (complementary metal oxide semiconductor) sensor. The CMOS sensor exhibits several advantages, including high sensitivity, low noise levels, and an extensive dynamic range. It demonstrates the capacity to capture high-quality images in the infrared light spectrum, thereby satisfying the demands of various applications, such as high-speed photography and accurate motion capture. This sensor was able to swiftly convert optical signals into electrical signals and perform digital processing, which enabled computers to analyze and manage these signals. As a result, it enabled the precise tracking and measurement of object movements. The analog signal collected during the test was converted into a digital signal by the data acquisition box and transmitted to the computer for the next step in data analysis. Before the experiment began, the motion capture system was used to eliminate or cover any reflective spots in the experimental area and to calibrate the experimental space. Using the upper and lower limb models from the Qualisys test system [14,15], 52 reflective markers were attached to the bodysuit. Participants were asked to perform the movement in a designated area for data collection. For static data collection, subjects stood with their feet naturally shoulder-width apart, facing forward, with forearms parallel to the ground, elbows bent, palms facing down, fingers naturally open, and eyes staring straight ahead for 10 s. Dynamic data acquisition was followed by removing the markers on the knee and ankle joints. For cases where some marker trajectories had missing data, the gaps were filled using Qualisys Track Manager (QTM 2023.1, Qualisys AB, Gothenburg, Sweden) based on the actual situation, using linear interpolation, correlation, and polynomial fitting methods.

### 2.5. Data Filtering and Pre-Processing

The preprocessed kinematic data (in C3D format) were imported into Visual 3D (V6.0, C-Motion, Germantown, MD, USA). A fourth-order Butterworth low-pass digital filter was applied to smooth the raw coordinate data, with a cutoff frequency for kinematics set at 14 Hz [15]. Anatomical landmarks and segments were defined based on the Visual 3D framework model and anthropometric data. This process involves specifying key anatomical points and segment definitions to accurately represent the body’s movement and structure within the model [16]. The COM position data were calculated using a 13-segment model and the weighted sum method [15]. Joint angles for the shoulder, elbow, wrist, trunk, head, hip, knee, and ankle were derived by calculating the angles between proximal and distal segments. This study primarily analyzed the joint angles in the coronal (X), sagittal (Y), and horizontal (Z) planes. The X–Y–Z Cardan sequence defines the rotation order based on the right-hand rule regarding the segment coordinate axes (Figure 2b).

### 2.6. Movement Phase Definition

**Figure 3:**
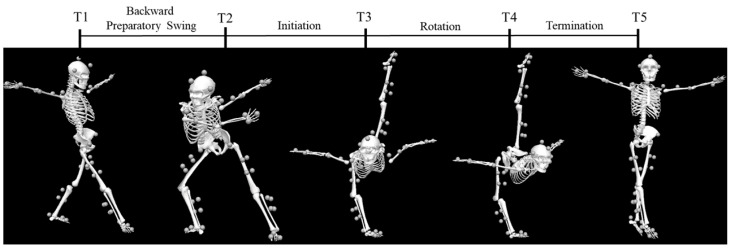
Movement phase definition (The circles represent marker clusters).

Based on the study’s needs, the vertical back split rotation in RG was divided into phases. Characteristic moments were defined based on specific features of the body segments. The entire vertical back split rotation was divided into four phases using the following five characteristic moments: the moment when the swinging leg (right leg) makes contact with the ground (T1), the moment when the preparatory swing reaches the body’s farthest left position (T2), the moment when the rotation begins (T3), the moment when the rotation ends (T4), and the moment when the movement ends (T5). The phases are as follows: backward preparatory swing phase (T1–T2), initiation phase (T2–T3), rotation phase (T3–T4), and termination phase (T4–T5) (Figure 3).

### 2.7. Statistical Analysis

Data were first organized and analyzed using Microsoft Excel 2016 (Microsoft Corporation, Redmond, WA, USA). The mean and standard deviation for certain angles were calculated using SPSS 25.0 (Statistical Package for the Social Sciences, version 25.0, IBM Corporation, New York, NY, USA). Trajectory plots for some of the data were created using Origin 2021 (Origin 2021 Graphing and Data Analysis Software, OriginLab Corporation, Northampton, MA, USA).

## 3. Results

Since the main movement of the C 807 is concentrated in the first three phases, we primarily investigated the COM trajectories during the backward preparatory swing, initiation, and rotation phases. According to the movement characteristics, the right leg serves as the support leg, while the left leg is defined as the swinging leg.

### 3.1. Temporal and Spatial Parameters of the Movement

The duration of the movement reflects both the speed at which the gymnast performs the action and the rhythm of the movement. The proportion of time spent in each phase of the entire movement process varies among athletes. Analysis of the kinematic data revealed the movement times and the duration of each phase for the eight gymnasts completing the C 807 (Table 1). The eight gymnasts’ total time to complete the movement ranged from 5.33 s to 6.8 s, with different phases occupying varying proportions of the total time. Overall, the initiation phase requires a quick push-off and, thus, occupies a smaller proportion of the total time. A shorter push-off time was observed to correlate with a faster push-off speed and a longer rotation time.

### 3.2. Joint Angle Characteristics at Characteristic Moments

Based on the movement, we divide it into five characteristic moments and four phases, studying the joint angles of both the upper and lower limbs. The changes in joint angles at different time points reflect adjustments in movement posture.

**Figure 4:**
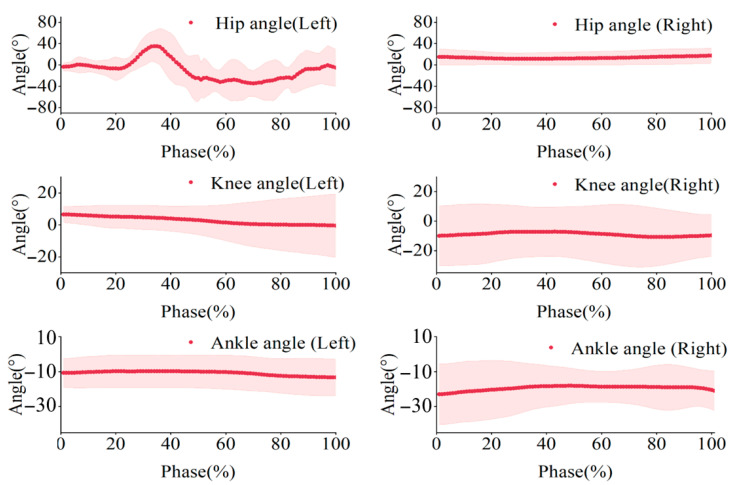
Mean joint angles of the hip, knee, and ankle. The shaded area indicates the standard deviation (SD) across participants.

For the support-side lower limb (Table 2), the sagittal plane angle of the hip joint gradually increases with hip flexion. The knee joint was flexed during the backward preparatory swing and initiation phases, but its angle remained almost unchanged during the rotation phase, which was in a state of hyperextension. The ankle joint was approximately 90°at moment T2, and its angle remained constant during the rotation phase. For the swinging-side lower limb (Table 3), the sagittal plane angle of the hip joint changed from positive to negative during the swing. The knee and ankle joint angles remained almost unchanged during the rotation phase. For the upper limb shoulder joints (Table 4 and Table 5), at moment T1, both arms were horizontally abducted. T2’s right arm had its shoulder and elbow joints flexed and internally rotated. In contrast, the left arm was abducted with a slight flexion of the elbow, preparing for the initiation phase. Throughout the rotation phase, both arms were slightly abducted, with slight changes in flexion angle. We used the entire movement cycle (T1–T4) as the normalized time scale (0–100%) and created the bilateral lower limb joint angle variation trajectories (Figure 4). We found that the hip joint angle changes significantly, while the knee and ankle joint angles remain almost unchanged.

Through the study of rotational movement, it is also worth examining the torsion of each limb segment, in addition to joint angles, during the backward preparing swing phase. A comparative study revealed that at moment T2, the tibia on the support side underwent significant rotation, with an angle of approximately 35.7° clockwise, which did not occur at other characteristic moments (Table 6). The trunk torsion angle was 37.2° at moment T2, with left and right hip torsion angles of 23.9° and 32.3°, respectively, indicating an overall reverse torsion, which prepared the gymnast for the initiation phase. The tibial rotation angle was the torsion angle between the support-side thigh and calf. The trunk rotation angle was the torsion angle between the trunk and pelvis. The hip joint rotation angle was the torsion angle between the pelvis and thigh.

### 3.3. Moving Trajectories of the COM in Different Stages

The center of mass (COM) represents the weighted average position of all mass segments in the human body. It is a significant indicator for evaluating the quality of completion in a sports action, analyzing its technical characteristics, and correcting any technical errors. Due to the different shapes of the human body, the length of the limbs, the height, and weight all affect the position of the COM, so in the process of sports technology analysis, whether for a dynamic action or static posture, the human COM parameter body is significant in the analysis of sports action. We primarily analyzed the first three phases of the vertical back split rotation.

**Figure 5:**
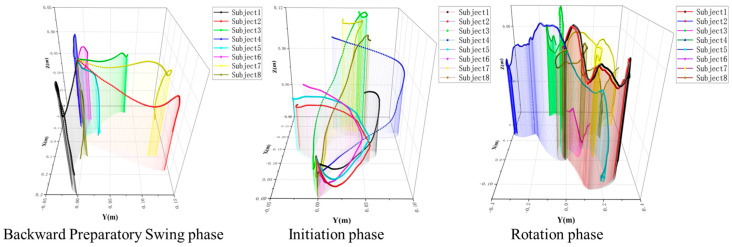
The trajectory of the COM changes in each phase. Each curve represents the trajectory of a subject at this stage. The starting point of the motion trajectory of 8 subjects was set as the origin of the coordinate system. X: the motion path in the forward and backward direction; Y: the motion path in the left and right direction; Z: the trajectory of motion in the up and down direction.

During the backward preparatory swing phase, the COM height generally decreased and changed as the body tilted, with a slight increase as it approached the initiation phase. The initiation phase exhibited a more pronounced effect, with the COM height initially decreasing and then increasing. The COM changed from large to small and then back to large, but almost all other positions, except height, remained unchanged. Although the COM height fluctuated in the rotation phase, it remained relatively constant. The more the COM swung in the horizontal plane, the worse the balance (Figure 5).

## 4. Discussion

Overuse injuries are quite common among competitive rhythmic gymnasts, with an estimated weekly prevalence rate of 37% [17]. The knees, lower back, and hip/groin are the most common injury locations [18]. Therefore, conducting a kinematic analysis of specific movements of rhythmic gymnasts is of great significance. This analysis accurately obtained movement parameters, which will help to optimize the proficiency of athletes’ technical movements and to effectively reduce the risk of injury during training and competitions. It should be noted that the difference between the mean and SD for some wrist angles is large because wrist action is not usually the primary focus of C 807, a technical movement in scoring, as well as due to the fact that elite athletes have individual differences in upper limb movements when completing technical movements. In our study, the C 807 in RG could be divided into three phases, namely the backward preparatory swing, initiation, and rotation phases, with the latter being the most critical. During the rotation phase, the support leg’s angle remained constant, providing stability, while the knee joint entered a state of hyperextension. This hyperextension enhanced the visual appeal of the movement by elongating the leg line, a key aesthetic requirement in RG. The hyperextension typically became more pronounced toward the end of the rotation due to the combined effects of inertia and centrifugal force acting on the support leg. However, this movement’s biomechanical demands could present risks. Excessive dorsiflexion during rotation could damage the ankle joint and reduce performance [19]. Similarly, excessive knee hyperextension and increased joint load can make the knee more susceptible to injury [20]. These risks highlighted the importance of athletes possessing substantial flexibility, leg strength, and core stability to execute movements effectively and safely. Performance assessments demonstrated that flexibility, leg strength, and visual–motor coordination were significantly correlated with RG outcomes [21]. Compared to the general population, RG athletes exhibit greater ranges of motion in the hip, knee, and ankle joints, accompanied by increased joint torques [22]. Additionally, their coordination, dynamic balance, and static balance scores were markedly higher than average [23]. These findings underscore the importance of targeted training in optimizing performance while minimizing injury risks.

A comparison of joint torsion angles between the backward preparatory swing phase and the initiation phase revealed consistently larger torsion angles at the hip joint. This was consistent with findings that effective rotational movements primarily occur at the hip [24]. In contrast, the knee joint torsion angle has to remain smaller to minimize the risk of injury during these movements [25]. At time T2, the supporting leg’s tibial torsion angle increased, resulting in external rotation of the supporting calf. Simultaneously, the swinging leg and the supporting hip joint rotated in opposite directions, facilitating the transition into the initiation phase. These coordinated movements highlighted the critical role of joint angles in optimizing technique while maintaining stability and preventing injury.

Successfully executing rotational movements required appropriate angular momentum, which depended on two key factors, namely the moment of inertia (MOI) and angular velocity. The MOI reflects the mass distribution relative to the axis of rotation; a more significant MOI makes it more challenging to alter the rotational state. In RG, gymnasts adjust their MOI by changing body posture. At the same time, angular velocity—how quickly an object rotates around its axis—determines the rotation speed [26]. Previous studies have shown that modifying body shape significantly affects the magnitude of rotational movements [27]. When angular momentum is insufficient to complete a rotation, adjustments in body posture, particularly involving the upper limbs, help facilitate the movement. For turns requiring more than three revolutions, lowering the arms and legs brings the body’s mass closer to the rotational axis, reducing the MOI and increasing rotational speed [28]. Skilled athletes enhance vertical angular momentum by generating momentum efficiently. They employ strategies, such as twisting the trunk relative to the pelvis during preparation and coordinating the arms to maximize angular momentum generation. The trailing arm is pivotal, contributing significantly to angular momentum during the double-stance phase [29]. In the rotation phase, keeping the swinging leg close to the vertical axis minimizes rotational inertia, resulting in smoother and more efficient rotation. Extended arms create a longer moment arm during the backward preparatory swing and initiation phases, generating greater torque and enhancing angular momentum. As the movement progresses, gymnasts flex their arms to increase angular velocity and maintain control. This interplay between arm position, ground reaction forces, and moment arms has a critical influence on the rotational speed and magnitude of torque [30]. To achieve a practical and aesthetically pleasing rotation, gymnasts must focus on extending their arms during the preparatory phases while leveraging tibial torsion in the supporting leg and counter-torsion in other lower limb joints to maximize rotational speed. During the rotation phase, the supporting leg must remain stable, the swinging leg should be close to the axis, and the arms should be slightly flexed to reduce rotational inertia and enhance efficiency. Integrating biomechanics and posture control is crucial for executing the movement precisely and elegantly.

Variations in joint angles, particularly those of the trunk and the sagittal plane angles of the hip, knee, and ankle, affect the height of the center of mass (COM), which is a key determinant of movement stability. Athletes lower their COM over a short interval to compress the body and store elastic potential energy, enabling efficient force generation for subsequent movements. During the backward preparatory swing phase, COM height is lowered through coordinated hip flexion, knee flexion, and ankle dorsiflexion. This compression increases elastic potential energy in preparation for the rotational initiation. During the initiation phase, changes in COM height are primarily driven by fluctuations in trunk and knee joint angles. Early in the phase, forward trunk lean reduces the COM height, followed by an increase in height as the knees extend in the latter part. The study highlights the functional relationship between trunk and hip flexion, where the hip extensors play a critical role in controlling trunk movement [31,32]. Training that focuses on strengthening the glutes and hamstrings can enhance control over these movements, thereby improving overall performance. In the rotation phase, the supporting knee joint remained hyperextended, while the trunk and ankle joint have minimal influence on COM height. Thus, flexibility in the knee and hip joints is crucial for making effective COM adjustments, which in turn contribute to the precision and stability of the movement. These findings underscore the importance of targeted flexibility and strength training in optimizing performance.

## 5. Conclusions

This study focused on the kinematic analysis of RG’s vertical post-split leg rotation, emphasizing the importance of joint angle rationality and standardization for proper execution. Ensuring precise joint angles is crucial for both improving movement quality and reducing injury risks. Training must prioritize the athlete’s leg swing and extension mechanics to improve performance and prevent injuries. Increasing the speed and strength of the swinging leg while ensuring that the supporting leg provides stability during rapid postural transitions is crucial. Additionally, optimizing the swing phase involves briefly lowering the COM to store muscle elastic potential energy. This requires greater hip and knee flexibility, as well as robust muscle strength around these joints. Incorporating knee joint stability exercises into training regimens is strongly recommended to address the risk of hyperextension injuries during rotation. Monitoring lower limb joint loads is essential to safeguarding athlete health. Furthermore, upper limb rhythm control can be enhanced through coordination exercises that target flexion and extension, thereby improving rotational balance and speed. By focusing on these biomechanical aspects, athletes could achieve better performance outcomes while reducing the risk of injury.

## Figures and Tables

**Table 1:** Time distribution table for each action phase (s).

	Backward Preparatory Swing Phase (T1–T2)	Initiation Phase (T2–T3)	Rotation Phase (T3–T4)	Termination Phase (T4–T5)	Total
Subject1	1.99	1.15	2.27	0.92	6.33
Subject2	1.03	1.08	2.29	0.95	5.35
Subject3	1.70	0.92	2.34	1.08	6.04
Subject4	0.67	1.00	3.65	1.03	6.35
Subject5	0.75	1.15	2.71	0.72	5.33
Subject6	0.76	1.04	3.36	1.09	6.25
Subject7	1.22	0.86	3.62	1.10	6.8
Subject8	1.23	0.87	3.25	1.01	6.36

**Table 2:** Left lower limb flexion/extension joint angle (°, M ± SD).

	Hip		Knee		Ankle	
	*X*-Axis	*Y*-Axis	*X*-Axis	*Y*-Axis	*X*-Axis	*Y*-Axis
T1	−15.1 ± 9.8	−1.4 ± 3.6	4.7 ± 4.6	2.2 ± 3.9	83.3 ± 10.4	12.5 ± 7.4
T2	54.0 ± 11.8	18.0 ± 3.7	14.1 ± 4.0	5.8 ± 4.3	58.9 ± 4.2	9.1 ± 5.1
T3	−47.0 ± 12.6	36.1 ± 6.8	−4.7 ± 5.9	5.2 ± 3.9	25.9 ± 7.6	2.0 ± 5.4
T4	−47.4 ± 13.2	36.6 ± 7.2	−3.2 ± 8.0	4.5 ± 3.2	25.9 ± 7.4	1.8 ± 4.8
T5	15.5 ± 17.3	−2.3 ± 6.7	4.8 ± 5.8	3.4 ± 3.1	48.1 ± 17.1	6.8 ± 8.0

**Table 3:** Right lower limb flexion/extension joint angle (°, M ± SD).

	Hip		Knee		Ankle	
	*X*-Axis	*Y*-Axis	*X*-Axis	*Y*-Axis	*X*-Axis	*Y*-Axis
T1	46.0 ± 9.1	−8.6 ± 6.4	−18.0 ± 6.1	1.8 ± 4.3	51.1 ± 13.2	−11.6 ± 8.7
T2	81.6 ± 11.7	−34.9 ± 11.1	−49.8 ± 8.9	14.5 ± 8.2	88.7 ± 8.1	−12.0 ± 11.2
T3	104.3 ± 16.5	−36.5 ± 8.2	22.4 ± 5.5	−6.6 ± 4.1	53.9 ± 6.2	−4.4 ± 5.7
T4	116.8 ± 21.7	−37.3 ± 6.2	24.8 ± 7.5	−8.2 ± 4.0	58.1 ± 6.2	−9.5 ± 5.6
T5	8.6 ± 12.1	−3.31 ± 3.1	4.6 ± 8.7	−3.9 ± 4.9	46.7 ± 8.1	−3.3 ± 6.0

**Table 4:** Left upper limb flexion/extension joint angle (°, M ± SD).

	Shoulder		Elbow		Wrist	
	*X*-Axis	*Y*-Axis	*X*-Axis	*Y*-Axis	*X*-Axis	*Y*-Axis
T1	5.4 ± 4.4	69.4 ± 11.5	7.6 ± 9.1	7.1 ± 6.6	−2.0 ± 12.3	−13.0 ± 13.6
T2	−73.8 ± 4.3	54.8 ± 16.9	6.2 ± 6.0	5.8 ± 6.5	2.3 ± 9.4	−3.0 ± 12.2
T3	−34.5 ± 11.0	59.0 ± 8.4	2.8 ± 9.4	4.5 ± 11.6	−8.3 ± 12.1	−5.6 ± 9.9
T4	−30.7 ± 9.6	45.5 ± 21.2	3.6 ± 5.4	5.0 ± 9.3	−1.6 ± 12.0	−3.1 ± 12.9
T5	6.8 ± 5.9	51.7 ± 20.0	8.0 ± 12.3	8.0 ± 10.2	−9.8 ± 10.7	−5.0 ± 11.3

**Table 5:** Right upper limb flexion/extension joint angle (°, M ± SD).

	Shoulder		Elbow		Wrist	
	*X*-Axis	*Y*-Axis	*X*-Axis	*Y*-Axis	*X*-Axis	*Y*-Axis
T1	8.1 ± 14.9	−68.6 ± 10.2	−3.6 ± 8.4	−6.3 ± 9.6	12.2 ± 12.1	3.4 ± 9.3
T2	93.1 ± 18.9	29.7 ± 10.0	82.4 ± 22.2	−1.2 ± 5.6	4.9 ± 12.7	−0.4 ± 11.4
T3	−32.5 ± 12.8	−41.0 ± 27.5	1.8 ± 14.6	−6.8 ± 10.2	0.9 ± 15.4	1.1 ± 10.7
T4	−35.2 ± 8.4	−30.3 ± 21.0	3.2 ± 10.4	−8.2 ± 12.7	5.1 ± 16.8	−1.5 ± 12.2
T5	6.4 ± 14.9	−41.2 ± 26.5	7.3 ± 8.8	5.6 ± 9.2	3.9 ± 21.7	−3.0 ± 14.0

**Table 6:** Helical angles at different phases’ angles (°, M ± SD).

	T1	T2	T3	T4	T5
Left hip joint torsion angle	−8.8 ± 7.0	23.9 ± 5.9	28.3 ± 4.3	28.5 ± 8.2	−3.2 ± 5.0
Right hip joint torsion angle	20.1 ± 6.2	32.3 ± 4.0	−4.7 ± 2.0	18.3 ± 13.7	−4.5 ± 3.4
Left tibial torsion angle	0.7 ± 25.1	−3.6 ± 8.7	−0.6 ± 8.5	−3.9 ± 2.7	−13.7 ± 7.1
Right tibial torsion angle	11.7 ± 2.8	35.7 ± 1.5	3.3 ± 5.0	3.7 ± 5.2	−7.3 ± 6.8
Trunk torsion angle	−12.0 ± 8.2	37.2 ± 4.5	−16.6 ± 7.5	−1.6 ± 4.9	−7.3 ± 6.6

## Data Availability

The data presented in this study are available on request from the corresponding author. The data are not publicly available due to confidentiality.
